# The Accuracy and Sensitivity of Delta Neutrophil Index in Malignancy: Diagnostic Study of Different Types

**DOI:** 10.3390/diagnostics15243187

**Published:** 2025-12-13

**Authors:** Hüseyin Emre Tepedelenlioğlu, Hüseyin Bilgehan Çevik, Özgen Ahmet Yildirim, Ahmet Kürşat Güneş, Erkan Akgün, Hanife Avcı

**Affiliations:** 1 Department of Orthopedics and Traumatology, Ankara Etlik City Hospital, Ankara 06170, Türkiye; bilgehancevik@gmail.com; 2 Department of Oncology, Ankara Etlik City Hospital, Ankara 06170, Türkiye; ozgenayildirim@gmail.com; 3 Department of Hematology, Ankara Etlik City Hospital, Ankara 06170, Türkiye; ahmetkgunes@gmail.com; 4 Department of Orthopedics and Traumatology, Lokman Hekim University, Ankara 06530, Türkiye; drorthopedic.akgun@gmail.com; 5 Department of Biostatistics, Hacettepe University, Ankara 06230, Türkiye; hanife.avci@hacettepe.edu.tr

**Keywords:** delta neutrophil index, immature granulocytes, hematologic malignancy, sarcoma, carcinoma, melanoma, diagnostics, pre-biopsy triage

## Abstract

**Background/Objectives:** The delta neutrophil index (DNI)—a hematology analyzer-derived measure of circulating immature granulocytes—may assist pre-biopsy decision-making, yet its behavior across tumor types is incompletely defined. We examined whether pre-biopsy DNI differs by pathology category, tumor class, and definitive histology, and evaluated diagnostic performance. **Methods:** In this retrospective, single-center cohort, consecutive inpatients with malignancy were screened (*n* = 2009). Exclusions included positive blood cultures, prior chemotherapy/radiotherapy before index labs, and lack of definitive pathology, yielding 1313 analyzable cases. All laboratories, including DNI, were obtained before diagnostic biopsy. DNI was assessed as a continuous variable and categorized (Zero = 0; High > 0.6). Groupwise differences used Kruskal–Wallis and χ^2^ tests with FDR control; discrimination used ROC analyses (one-versus-rest/pairwise). **Results:** DNI distributions differed across pathology, tumor class, and definitive diagnoses (all *p* < 0.001). High DNI (>0.6) and Zero DNI (=0) proportions also varied significantly by grouping. Hematologic malignancies showed the highest DNI (median ~1.0) compared with sarcoma and carcinoma (medians ~0.4). Using DNI alone, one-versus-rest AUCs were 0.735 (hematologic), 0.692 (melanoma), 0.672 (sarcoma), and 0.652 (carcinoma); the strongest pairwise separation was hematologic versus sarcoma (AUC 0.780). For specific solid tumors, including breast and renal cell carcinoma, single-marker discrimination was modest; no clinically actionable RCC cutoff emerged. Sensitivity analyses restricted to culture-negative cases yielded consistent findings. **Conclusions:** Pre-biopsy DNI exhibits tumor-type-dependent variation and provides adjunct diagnostic signal—the strongest for hematologic malignancy—yet is insufficient alone for solid tumor subtyping. Integration with clinical assessment and routine biomarkers, and multi-center validation with device harmonization are warranted.

## 1. Introduction

Early and accurate cancer diagnosis is critical for timely treatment selection and improved outcomes. In orthopedic oncology, bone and soft-tissue tumors often present with non-specific symptoms such as pain, swelling, and clinical overlap with benign lesions, which frequently delays the diagnosis and treatment, and adversely affects prognosis [1]. In this context, simple blood-based markers that are available at first presentation and can refine pre-test probabilities before biopsy are attractive adjuncts to imaging and clinical assessment. Accordingly, rigorous clinical assessment, appropriate imaging, and timely biopsy are essential to ensure diagnostic accuracy and therapeutic success; delays may permit tumor progression, narrow therapeutic options, and shorten survival, while misdiagnosis can lead to unnecessary procedures and increased healthcare costs [2,3].

Patients presenting to orthopedic oncology commonly report pain arising from bone or soft-tissue lesions, swelling, restricted range of motion, or pathologic fractures; age distribution is broad with peaks in childhood and later adulthood, and sex distribution is typically balanced. The malignant spectrum often includes metastatic disease; predominantly of pulmonary and hematologic origin, as well as primary entities such as osteosarcoma, chondrosarcoma, giant cell tumor, and liposarcoma. Health system factors (referral pathways, socioeconomic status, and insurance coverage) can further influence time-to-diagnosis and access to definitive care [1,4,5].

Tumor biomarkers reflect both neoplastic biology and host responses to tumor development and progression. Classical analytes (e.g., AFP, NSE, CEA, CA19-9, CA15-3, CA125) may rise in selected histologies but lack universal specificity [6]. Systemic inflammatory markers such as C-reactive protein (CRP) and erythrocyte sedimentation rate (ESR) are frequently elevated in malignancy, yet they also respond to trauma and infection, limiting diagnostic precision [7,8]. Neutrophils are key cellular mediators of tumor-associated inflammation: tumor-derived cytokines and growth factors promote expansion and activation of neutrophil and immature myeloid populations, which in turn can support tumor growth, angiogenesis, and immune evasion [9]. The degree of neutrophil activation and bone-marrow myelopoiesis thus varies across malignancy types and may be reflected in peripheral blood indices. These observations provide a biological rationale for evaluating DNI, as a surrogate of neutrophil/immature granulocyte activity, in patients with suspected malignancy.

Immature granulocytes (IG) emerge early during bone-marrow activation, prior to overt neutrophilia, and have been explored as adjunctive markers across diverse diseases [10]. The delta neutrophil index (DNI), a hematology analyzer-derived parameter quantifying circulating immature granulocytes by comparing leukocyte differentials measured in dedicated optical channels, is reported automatically as part of a routine complete blood count. Elevated DNI has been investigated in selected cancers, including thyroid and breast, and with the presence of endometrial and renal cell carcinoma, suggesting that DNI can differentiate between selected cancer types and benign controls. However, comprehensive tumor-type-wide evaluations at the pre-biopsy stage remain scarce [11,12].

We therefore conducted a retrospective, single-center analysis of consecutive patients managed by orthopedic oncology to assess whether pre-biopsy DNI differs across pathology categories, tumor classes, and definitive histologic diagnoses. To minimize infection-related confounding, culture-positive cases were excluded. We hypothesized that DNI would demonstrate variation by malignancy type and specific tumor groups would exhibit characteristic DNI patterns, supporting its use as an adjunct diagnostic signal prior to tissue diagnosis.

## 2. Materials and Methods

### 2.1. Study Design, Setting, and Data Sources

This was a retrospective, single-center cohort study. Electronic records were retrieved from the Hospital Information System and the Laboratory Information System and reviewed retrospectively.

### 2.2. Participants and Eligibility

Consecutive inpatients aged 17–99 years, irrespective of sex, who were admitted for treatment with a diagnosis of malignancy were screened (*n* = 2009). Primarily, the included patients admitted to our orthopedic oncology clinic had bone metastases or primary bone and soft-tissue sarcoma. The following were excluded (*n* = 696): patients whose malignancy did not involve either bone or soft-tissue metastasis or a primary bone/soft-tissue sarcoma (i.e., patients outside the orthopedic oncology case-mix), patients who had received chemotherapy and/or radiotherapy at an outside center before the index laboratory assessment, and patients with positive blood culture results. After exclusions, 1313 patients comprised the analytic cohort.

### 2.3. Variables and Timing of Measurements

The following variables were abstracted: age, sex, tumor stage, pathology diagnosis, and pre-procedure laboratory values obtained before any operative intervention/diagnostic biopsy. Laboratory parameters included DNI, CRP, GGT, AST, ALT, LDH, AFP, ALP, direct bilirubin, indirect bilirubin, PT, APTT, INR, procalcitonin (PCT), CA19-9, CEA, CA125, WBC, lymphocyte percentage, and neutrophil percentage. Each laboratory value was additionally categorized as high, normal, or low according to the laboratory reference ranges indicated in the dataset headers (CRP < 5, GGT < 40, AST < 40, ALT < 41, LDH < 232, AFP < 7, ALP: 40–129, direct bilirubin < 0.35, indirect bilirubin: 0.3–1, PT: 8.4–10.6, APTT: 23.6–33.2, INR: 0.9–1.27, PCT < 0.5, CA19-9 < 34, CEA < 5, CA15-3 < 28, CA125 < 5). DNI was obtained automatically from the Siemens ADVIA 2120 hematology analyzer used in our laboratory. The analyzer calculates DNI as the difference between leukocyte differentials measured in the myeloperoxidase (MPO) channel and in the nuclear lobularity channel, corresponding to the relative proportion of circulating immature granulocytes; results are reported as a percentage of neutrophil-related cells [13]. In routine practice at our center, values from 0.0% to 0.6% are considered within the manufacturer-recommended reference interval. Accordingly, for categorical analyses we defined three DNI groups: ‘Zero’ (exactly 0.0%), ‘Non-zero within reference’ (0.1–0.6%), and ‘High’ (>0.6%, above the upper limit of the local reference range). The >0.6% threshold thus reflects the local upper reference limit rather than a data-driven optimum derived from this cohort.

### 2.4. Statistical Analysis

Analyses were performed using the free and open-source software R (version 4.4.1, https://cran.r-project.org), and SPSS for Windows Version 23.0 statistical package (Chicago, IL, USA) by an academic biostatistician. Normality of the data was assessed using the Kolmogorov–Smirnov test, and variance homogeneity was tested using Levene’s test. Descriptive statistics were presented as median (25th percentile–75th percentile) and frequencies (percentages) as appropriate. To compare differences between groups, the non-parametric Kruskal–Wallis test was used for continuous variables, and Pearson chi-square test was used for categorical variables. Dunn’s Bonferroni test was used as the post hoc test following the Kruskal–Wallis test. For the non-parametric Kruskal–Wallis test, the effect size was estimated using epsilon-squared (*ε*2) and Cramer’s V for the Pearson chi-square test.

To evaluate the effect of the DNI as a potential risk factor across tumor classifications, a multinomial logistic regression analysis was performed. To evaluate the effect of the DNI as a potential risk factor across tumor classifications, a multinomial logistic regression analysis was performed. Sarcoma was defined as the reference category, because primary bone and soft-tissue sarcomas represent the central diagnostic concern in orthopedic oncology, and we sought to quantify how changes in DNI shift the relative odds of a lesion being classified as hematologic malignancy, carcinoma, or melanoma instead of primary sarcoma. In addition, sarcoma cases are likely to have better prognosis and earlier stage diagnosis, unlike metastatic tumors admitted to orthopedic oncology clinics. Odds ratios (ORs) with 95% confidence intervals (CIs) were reported for hematologic, carcinoma, and melanoma groups. Violin plots and forest plots were generated using the “ggplot2” package [14].

To assess the discriminative ability of the DNI across the four tumor categories (hematologic, carcinoma, melanoma, and sarcoma), we fitted a support vector machine classifier with a polynomial kernel (SVM-poly) using DNI as the predictor. Multiclass performance was summarized by receiver operating characteristic (ROC) analysis. Area under the curve (AUC) values were computed using two schemes: one-versus-one (OvO), yielding an AUC for each pairwise class comparison, and one-versus-rest (OvR), yielding an AUC for each class against all remaining classes. All analyses were performed in R version 4.5.1. using the following packages: “readxl” [15], “caret” [16], “pROC” [17], “dplyr” [18], and “e1071” [19]. The statistical significance level was accepted as *p* < 0.05.

## 3. Results

Demographic data are presented in Table 1. Fifty-eight percent were male (*n* = 762) and forty-two percent were female (*n* = 551). The median age was 63 years (range, 53–70). Median values of key laboratory parameters were as follows: C-reactive protein (CRP) 19.23 mg/L (5.05–58.40), neutrophil-to-lymphocyte ratio (NLR) 0.4 (0.3–0.8), and white blood cell (WBC) count 8.05 × 10^9^/L (5.97–10.52). The distribution of tumor types was carcinoma 62.9% (*n* = 826), hematologic malignancies 27.5% (*n* = 361), sarcoma 8.6% (*n* = 113), and melanoma 1.0% (*n* = 13).

**Figure 1:**
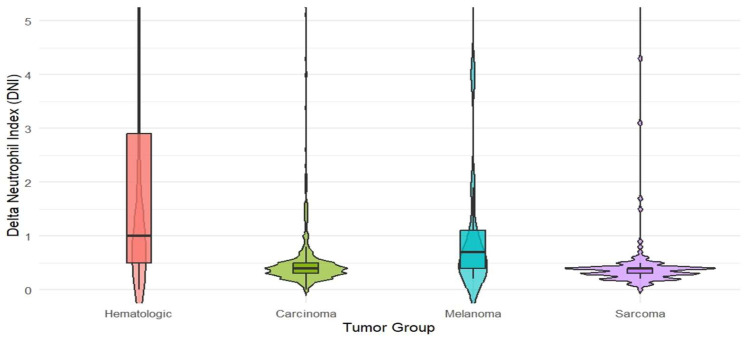
Violin plots of DNI (Delta Neutrophil Index) distributions by tumor class: hematologic malignancies (*n* = 361), carcinoma (*n* = 826), melanoma (*n* = 13), and sarcoma (*n* = 113). Central lines indicate group medians and interquartile ranges. (Between the four tumor classes, differences in DNI values were examined using the Kruskal–Wallis test).

In the comparison between groups, statistically significant differences were found for age (*p* < 0.001; η^2^ ≈ 0.045), C-reactive protein (CRP) (*p* < 0.001; η^2^ ≈ 0.025), DNI (Delta Neutrophil Index) (*p* < 0.001; η^2^ ≈ 0.151), and procalcitonin (*p* < 0.001; Cramer’s V ≈ 0.042) (Kruskal–Wallis/chi-square test). The median DNI was highest in the hematologic tumor group at 1.0 (0.5–2.9); in carcinoma, it was 0.4 (0.3–0.5), in sarcoma 0.4 (0.3–0.4), and in melanoma 0.7 (0.4–1.1) (Table 2). According to the categorical threshold, the frequency of DNI > 0.6 was 68.7% in the hematologic group, 18.4% in carcinoma, 53.8% in melanoma, and 8.0% in sarcoma (*p* < 0.001; V ≈ 0.500). These distributions are illustrated with violin plots in Figure 1.

Significant differences in DNI levels were observed among hematologic subtypes (*p* < 0.001); the highest median value was found in CML (chronic myeloid leukemia) at 19.9 (range 5.4–30.8). Median values in ALL/AML (acute lymphoblastic/acute myeloid leukemia) were 1.7, Hodgkin/NHL (non-Hodgkin lymphoma) 0.7, CLL (chronic lymphocytic leukemia) 0.4, and multiple myeloma 1.0. Significant differences were also noted among carcinoma subtypes (*p* < 0.001); for example, the median DNI in prostate cancer was relatively elevated at 0.8 (0.5–2.1), whereas in other subtypes it mostly ranged between 0.2 and 0.7 (Table 3).

**Figure 2:**
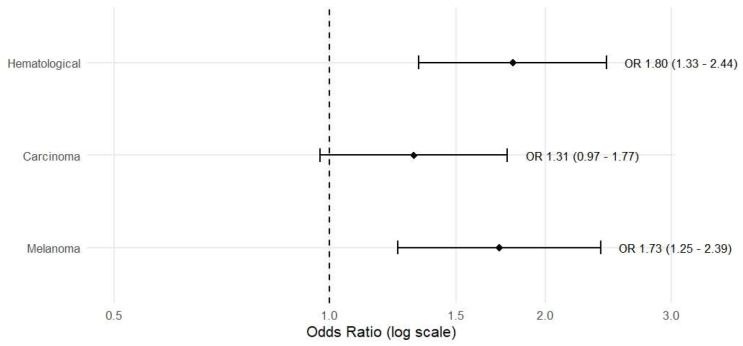
Forest plot of odds ratios (ORs) and 95% confidence intervals for the association between DNI (per 1-unit increase) and tumor class, obtained from multinomial logistic regression using sarcoma as the reference category. Bars show ORs for hematologic malignancies (*n* = 361), carcinoma (*n* = 826), and melanoma (*n* = 13); *p*-values are derived from the multinomial logistic regression model.

Using sarcoma as the reference category, each unit’s increase in DNI was associated with a 1.801-fold higher likelihood of belonging to the hematologic tumor group (95% confidence interval [CI] 1.332–2.436; *p* < 0.001), and similarly, a 1.725-fold increase for melanoma (95% CI 1.245–2.390; *p* = 0.001). PCT was not included in the risk model because it was not significant as a risk factor in the model. For carcinoma, the odds ratio (OR) was 1.310 (95% CI 0.970–1.771; *p* = 0.079), which did not reach statistical significance (Table 4). These results indicate that DNI provides stronger prior diagnostic information for hematologic and melanoma subgroups at the time of diagnosis, whereas additional evidence is needed for carcinoma (Figure 2).

In the multiclass discrimination performed using DNI as a single marker, pairwise (one-versus-one, OvO) AUC values ranged from 0.37 to 0.78; the most pronounced discriminations were observed between hematologic malignancies and sarcoma (AUC 0.780) and between melanoma and sarcoma (AUC 0.753) (Table 5). In the one-versus-rest (OvR) approach, AUCs were 0.735 for hematologic cancers, 0.692 for melanoma, 0.672 for sarcoma, and 0.652 for carcinoma, generally indicating a moderate level of discriminatory power. These findings support that DNI alone is not definitive for diagnosis but serves as a complementary tool alongside clinical and other laboratory data (Table 6).

## 4. Discussion

Early timing and precision of diagnosis in bone and soft-tissue tumors is challenging because presenting symptoms are often non-specific and may overlap with benign conditions. In this context, DNI has attracted interest as an adjunctive biomarker for diagnostic triage and risk stratification. Our study adds pre-biopsy, culture-negative evidence to this literature by showing that DNI varies meaningfully across tumor categories and specific histologies, and that both extreme categories, Zero (DNI = 0) and High (DNI > 0.6), carry information. The findings indicate that a DNI profile consistent with a higher inflammatory response is more prominent in hematologic malignancies, whereas lower levels predominate in solid tumors. Another notable observation is the significant difference in the distribution of cases with a DNI of zero among the classes; this suggests that not only “high” values but also “zero” values may carry biological and clinical significance.

DNI, an inexpensive and rapidly available parameter reflecting the circulating fraction of immature granulocytes, captures marrow activation that may be accentuated in hematologic malignancies due to altered myelopoiesis and non-infectious inflammatory stimuli. In solid tumors, the same response is more heterogeneous, influenced by tumor biology and host immune status. Consistent with this biology, our data show that a single pre-biopsy DNI measurement still carries incremental diagnostic signal at the group level (e.g., across tumor classes), even if subtype-level PPV within sarcomas remains modest. Practically, this supports using DNI as an adjunct to aid triage in the emergency or initial clinic setting, to flag higher-risk subgroups earlier, and to refine pre-test probabilities before tissue diagnosis—while reserving definitive decision-making for integrated assessment with imaging, pathology, and complementary biomarkers.

DNI offers a practical advantage in that it is generated automatically with the complete blood count and requires no additional calculations. Although other inflammatory markers, such as the neutrophil-to-lymphocyte ratio (NLR) and platelet-to-lymphocyte ratio (PLR) have demonstrated diagnostic and prognostic relevance, several studies have reported superior diagnostic performance for DNI compared with NLR and PLR [12,20]. Next-generation composite indices, including the systemic immune-inflammation index (SII) and prognostic nutritional index (PNI), also show promise, particularly in early-stage gastric and head and neck cancers. For example, PNI has achieved an AUC of up to 0.908 in gastric cancer [21], while an AUC of 0.688 has been reported in follicular lymphoma [22]. In our cohort, DNI yielded an AUC of 0.735 for discriminating hematologic malignancies, supporting its reliability as an adjunct diagnostic biomarker. Given the differences in endpoints, case mix, and analytic methods across studiaes, these cross-study comparisons should be interpreted cautiously; nevertheless, the aggregate evidence suggests that DNI provides a clinically useful signal and can complement established inflammatory indices in pre-biopsy risk stratification.

DNI has been reported to be significantly higher in malignant than benign cohorts in several tumor types, including renal cell carcinoma (RCC), endometrial cancer, breast cancer, and thyroid malignancies. In a study of 73 patients with RCC, the diagnostic accuracy of DNI was high (AUC = 0.894) with a proposed cutoff of 0.45 [20]. In our series, the corresponding performance was more modest (AUC = 0.408), a finding likely driven by the limited number of RCC cases in our cohort, which constrains discriminative power and precision of the estimate. In endometrial cancer, DNI values > 2.75% have been associated with an approximately 11-fold higher risk of malignancy [23]. In thyroid cancer, both DNI and the immature granulocyte (IG) count differentiate benign from malignant lesions with high discriminative performance (AUC = 0.847), and a cutoff of ≥0.35 favors malignancy [12]. In breast cancer, DNI and IG have demonstrated high sensitivity and specificity for predicting axillary metastasis [24].

To date, there are no studies directly evaluating the association between the DNI and sarcoma diagnosis or prognosis. In sarcomas, hematologic biomarkers most frequently investigated include the neutrophil-to-lymphocyte ratio (NLR), platelet-to-lymphocyte ratio (PLR), and the systemic immune-inflammation index (SII) [25]. Large-scale analyses in soft-tissue and retroperitoneal sarcomas have demonstrated that elevated NLR is associated with poor prognosis, higher metastatic risk, and reduced overall survival. Moreover, NLR has emerged as an independent prognostic factor in specific subtypes such as Ewing sarcoma and undifferentiated pleomorphic sarcoma [26]. In our series of 113 sarcoma patients, the most frequently evaluated subtypes—pleomorphic sarcoma, Ewing sarcoma, myxofibrosarcoma, liposarcoma, chondrosarcoma, and osteosarcoma—demonstrated limited positive predictive value (PPV) when using DNI for subtype-level discrimination We did not measure SII because there is no defined normal range yet. This finding indicates that, within sarcomas, a fixed DNI-based rule is insufficient as a standalone diagnostic tool and should be interpreted in conjunction with clinical and laboratory context.

From an analytical standpoint, classifying all laboratory parameters as ‘Normal’ or ‘High’ based on the parenthetical reference ranges provided in the dataset offers a practical approach to mitigating inter-laboratory variability. For DNI, the >0.6% threshold corresponds to the upper limit of the manufacturer-recommended reference interval on our analyzer and was chosen a priori, rather than being optimized on this dataset. Nevertheless, this categorical boundary may still be influenced by instrument-specific calibration and pre-analytical factors (e.g., timing of sampling). Accordingly, multi-center validation and, where appropriate, site-specific threshold optimization are recommended. Although excluding culture-positive cases was intended to minimize infection-related false elevations, the limited sensitivity of blood cultures means that occult infection cannot be completely ruled out. Importantly, the preservation of the DNI signal in our culture-negative sensitivity analyses supports the robustness of our observations.

PCT is primarily utilized for the diagnosis of infection and sepsis, with only a limited role in cancer diagnosis. In gynecologic malignancies such as vulvar cancer, PCT and C-reactive protein (CRP) may assist in gauging the inflammatory response and in excluding concomitant infection; however, their sensitivity and specificity for direct tumor detection are low [27]. Consistent with these reports, PCT showed no meaningful diagnostic or prognostic value for malignancy in our cohort. Taken together, PCT may improve diagnostic accuracy when used alongside DNI in settings where infection must be rigorously excluded, but it should not be relied upon as a standalone biomarker for cancer diagnosis.

This study has several limitations. First, its retrospective, single-center design is susceptible to sampling and measurement bias and precludes causal inference. Second, some histologic subgroups, notably melanoma (*n* = 13), plasmocytoma (*n* = 2), and chronic myeloid leukemia (CML; *n* = 10) were small, so the subgroup-specific medians and discrimination metrics should be regarded as exploratory and imprecise. Third, inter-instrument calibration differences and pre-analytical factors may limit the generalizability of any DNI threshold. Fourth, concomitant conditions (e.g., corticosteroid use, occult infection) could not be completely excluded. These limitations underscore the need for prospective, multi-center validation with cross-platform standardization.

## 5. Conclusions

DNI, measured in the pre-biopsy period, can provide an additional clue as a decision support tool, particularly in cases suggestive of hematologic malignancy. However, DNI should not be used as a standalone diagnostic tool; it must be interpreted in conjunction with clinical findings, imaging studies, and standard biomarkers. Prospective, multi-center studies are needed to clarify the role of DNI as an early-stage biomarker that adds value in multivariable clinical prediction models. Such studies should focus on inter-device harmonization, validation of thresholds specific to pathology types, and the prognostic/diagnostic significance of temporal changes in DNI.

## Figures and Tables

**Table 1:** Demographic and clinical characteristics of the participants (*n* = 1313).

**Variables**	**Participants (*n* = 1313)**
**Gender**	
Male	762 (58%)
Female	551 (42%)
**Age**	63 (53–70)
**CRP**	19.23 (5.05–58.40)
**DNI**	0.4 (0.3–0.8)
**GGT**	31 (18–66)
**AST**	20 (15–28)
**ALT**	15 (11–25)
**LDH**	229 (184–329)
**AFP**	2.23 (1.45–3.89)
**ALP**	94 (73–139)
**Direct bilirubin**	0.2 (0.14–0.29)
**Indirect bilirubin**	0.26 (0.17–0.41)
**PT**	9.40 (8.83–10.10)
**APTT**	27.5 (25.4–29.9)
**INR**	1.03 (0.97–1.11)
**PCT**	0.11 (0.06–0.25)
**CA19-9**	23.90 (8–99.50)
**CEA**	3.57 (1.71–15.50)
**CA15-3**	26.70 (16.70–49.55)
**CA125**	43.10 (14.60–152)
**WBC**	8.05 (5.97–10.52)
**Lymphocyte percentage**	22.80 (14.80–31.20)
**Neutrophil percentage**	65.1 (54.4–74.2)
**Tumor Classification**	
Hematological	361 (27.5%)
Carcinoma	826 (62.9%)
Melanoma	13 (1%)
Sarcoma	113 (8.6%)

Data are presented as median (25. percentile–75. percentile). Categorical variables reported as frequency (percent).

**Table 2:** Comparison of demographic and laboratory characteristics across patient groups.

Variables	Hematological (*n* = 361)	Carcinoma (*n* = 826)	Melanoma (*n* = 13)	Sarcoma (*n* = 113)	*p*-Value	Effect Size ($\varepsilon^{2}$)
**Age**	60 (45–69) a	64 (57–70) bc	66 (59–78) c	54 (38–65) d	<0.001 *	0.045
**CRP**	18.61 a (4.53–53.97)	22.51 ca (6.70–61.90)	5.51 abc (1.98–31.77)	6.83 b (2.69–34.18)	<0.001 *	0.025
**DNI**	1 a (0.5–2.9)	0.4 b (0.3–0.5)	0.7 abc (0.4–1.1)	0.4 cb (0.3–0.4)	<0.001 *	0.151
**Procalcitonin**	0.09 a (0.06–0.20)	0.13 b (0.06–0.30)	0.09 abc (0.05–0.19)	0.06 c (0.03–0.13)	<0.001 *	0.043
**DNI (Categoric)**						
≤ **0.6**	113 (31.3%)	674 (81.6%)	6 (46.2%)	104 (92%)		
**>0.6**	248 (68.7%)	152 (18.4%)	7 (53.8%)	9 (8%)	<0.001 **	0.500

Data are presented as median (25. percentile–75. percentile). Categorical variables reported as frequency (percent). Differences between groups were analyzed using the following: * Kruskal–Wallis test, ** Pearson chi-square test. Dunn–Bonferroni post hoc results are indicated by superscript letters (a, b, c, etc.). Superscript letters indicate the results of Dunn–Bonferroni post hoc comparisons. Groups that share at least one common letter do not differ significantly, whereas groups with no common letters differ significantly (*p* < 0.05).

**Table 3:** Pathologies of tumors.

*Pathology of Hematological Tumors (n = 361)*	DNI	*p*-Value
ALL (*n* = 27)	1.7 (0.8–4.5)	<0.001
AML (*n* = 115)	1.7 (0.9–5.8)	
Hodgkin Lymphoma (*n* = 28)	0.7 (0.4–1.1)	
CLL (*n* = 18)	0.4 (0.2–0.6)	
CML (*n* = 10)	19.9 (5.4–30.8)	
Multiple Myeloma (*n* = 57)	1 (0.7–3)	
Non-Hodgkin Lymphoma (*n* = 104)	0.7 (0.4–1)	
Plazmositoma (*n* = 2)	0.6 (0.4–0.7)	
***Pathology of carcinoma tumors (n = 826)***	**DNI**	***p*** **-value**
Pulmonary (*n* = 242)	0.4 (0.3–0.5)	<0.001
Skin (*n* = 7)	0.5 (0.3–0.5)	
Oral cavity (*n* = 8)	0.2 (0.2–0.3)	
Endometrium (*n* = 37)	0.4 (0.3–0.7)	
Pharyngeal (*n* = 15)	0.3 (0.3–0.5)	
Hepatocellular (*n* = 13)	0.5 (0.3–0.7)	
Colangiocellular (*n* = 16)	0.3 (0.2–0.5)	
Colorectal (*n* = 144)	0.4 (0.3–0.5)	
Laryngeal (*n* = 21)	0.3 (0.2–0.4)	
Breast (*n* = 114)	0.4 (0.3–0.5)	
Urothelial (*n* = 24)	0.5 (0.4–0.7)	
Gastric (*n* = 88)	0.4 (0.3–0.5)	
Over (*n* = 38)	0.4 (0.3–0.6)	
Oesophageal (*n* = 8)	0.4 (0.4–0.5)	
Pancreatic (*n* = 58)	0.3 (0.3–0.5)	
Parotideal (*n* = 4)	0.4 (0.2–0.4)	
Peritoneal (*n* = 4)	0.4 (0.4–0.4)	
Prostate (*n* = 28)	0.8 (0.5–2.1)	
Renal cell (*n* = 19)	0.3 (0.3–0.5)	
Cervix (*n* = 7)	0.4 (0.2–0.7)	
Thyroideal (*n* = 5)	0.5 (0.2–1)	

Data are presented as median (25. Percentile–75. percentile). *p*-values obtained using the Kruskal–Wallis test.

**Table 4:** Odds ratio comparisons between tumor types.

Tumor Classification	OR (95% CI)	*p*-Value
Hematological	1.801 (1.332–2.436)	<0.001
Carcinoma	1.310 (0.970–1.771)	0.079
Melanoma	1.725 (1.245–2.390)	0.001

The reference category is sarcoma. Abbreviations: OR: Odds ratio; CI: Confidence Interval.

**Table 5:** One-vs-One (OvO) approach.

Comparison	AUC *	Cut-Off	Sensitivity	Specificity
Hematologic vs Carcinoma	0.734	0.209	0.731	0.794
Hematologic vs. Melanoma	0.368	0.183	0.815	0.333
Hematologic vs. Sarcoma	0.780	0.196	0.778	0.879
Carcinoma vs. Melanoma	0.735	0.314	0.960	0.667
Carcinoma vs. Sarcoma	0.376	0.188	1.000	0.030
Melanoma vs. Sarcoma	0.753	0.017	0.667	0.939

* Abbreviations: AUC: Area under the curve.

**Table 6:** One-vs-Rest (OvR) approach.

Comparison	AUC *
Hematologic	0.735
Carcinoma	0.652
Melanoma	0.692
Sarcoma	0.672

* Abbreviations: AUC: Area under the curve.

## Data Availability

The original contributions presented in the study are included in the article, further inquiries can be directed to the corresponding author.
